# Non-Operative Management of Patients with Rectal Cancer: Lessons Learnt from the OPRA Trial

**DOI:** 10.3390/cancers14133204

**Published:** 2022-06-30

**Authors:** Paolo Goffredo, Felipe F. Quezada-Diaz, Julio Garcia-Aguilar, J. Joshua Smith

**Affiliations:** 1 Department of Surgery, Memorial Sloan Kettering Cancer Center, New York, NY 10065, USA; goffrep@mskcc.org (P.G.); garciaaj@mskcc.org (J.G.-A.); 2 Colorectal Unit, Department of Surgery, Complejo Asistencial Doctor Sótero del Río, Santiago 8150215, Chile; ffquezad@gmail.com

**Keywords:** neoadjuvant therapy, rectal cancer, total neoadjuvant therapy, chemoradiation, nonoperative management, watch and wait

## Abstract

**Simple Summary:**

The management of rectal cancer has become increasingly more complex. Over the recent year, the use of chemotherapy and radiation before surgical intervention has been accepted as a new standard. As a consequence, between a third and half of the patients undergoing upfront therapy experience a clinical complete response with no residual rectal tumor remaining in the bowel lumen. These patients could potentially avoid the risks of surgery and undergo a close surveillance protocol, known as watch and wait. However, the identification of ideal candidates for this strategy remains challenging due to the lack of objective criteria. Ongoing studies are investigating optimal treatment algorithms to further expand the indications for watch and wait.

**Abstract:**

Over the past decade, the management of locally advanced rectal cancer (LARC) has progressively become more complex. The introduction of total neoadjuvant treatment (TNT) has increased the rates of both clinical and pathological complete response, resulting in excellent long-term oncological outcomes. As a result, non-operative management (NOM) of LARC patients with a clinical complete response (cCR) after neoadjuvant therapy has gained acceptance as a potential treatment option in selected cases. NOM is based on replacement of surgical resection with safe and active surveillance. However, the identification of appropriate candidates for a NOM strategy without compromising oncologic safety is currently challenging due to the lack of an objective standardization. NOM should be part of the treatment plan discussion with LARC patients, considering the increasing rates of cCR, patient preference, quality of life, expectations, and the potential avoidance of surgical morbidity. The recently published OPRA trial showed that organ preservation is achievable in half of rectal cancer patients treated with TNT, and that chemoradiotherapy followed by consolidation chemotherapy may an appropriate strategy to maximize cCR rates. Ongoing trials are investigating optimal algorithms of TNT delivery to further expand the pool of patients who may benefit from NOM of LARC.

## 1. Introduction

Historically, the standard of care for locally advanced rectal cancer (LARC) has been neoadjuvant long-course chemoradiotherapy (LCRT), followed by total mesorectal excision (TME) and postoperative adjuvant chemotherapy [1]. However, starting in 2018, the National Comprehensive Cancer Network guidelines included in their recommendations the option for Total Neoadjuvant Therapy (TNT) with the administration of systemic chemotherapy in the neoadjuvant settings [1]. This change reflects the increasing complexity of the treatment algorithms for LARC observed over the past decade, balancing the goals of achieving better outcomes with improving quality of life (QoL) [2].

It is well established that a proportion of LARC patients receiving neoadjuvant therapy (NAT) experience pathological complete responses (pCR), defined as the absence of residual tumor cells at the primary tumor site and the mesorectal lymph nodes [3]. While ~20% of LARC patients undergoing LCRT alone have a pCR, the rate of complete responders may be as high as 40–60% with the implementation of TNT regimens [4]. Patients achieving pCR demonstrate excellent survival, with fewer than 5% of systemic recurrence and 1% of local failure [3].

However, TME significantly impacts patients’ QoL, with reported rates of bowel, genitourinary, and sexual dysfunction ranging from 30 to 80% [5,6]. Therefore, due to the high proportion of tumor response associated with novel NAT modalities, the benefit of TME in patients achieving a complete response has been questioned, resulting in several investigations of a non-operative management (NOM) strategy for patients with a clinical complete response (cCR). Notwithstanding the major challenges involving the appropriate identification of patients with cCR, NOM for LARC continues to gain acceptance as a potential treatment option for selected patients given the potential benefits of avoiding radical surgery.

The aims of the current review were to discuss the principles of NOM of LARC and provide a comprehensive update of the recent trials on the topic, highlighting investigator perspectives and insights from the Organ Preservation of Rectal Adenocarcinoma (OPRA) trial with a focus on the selection of patients appropriate for NOM.

## 2. Overview of Neoadjuvant Therapy

In the classic paradigm for the treatment of patients with LARC, consisting of chemoradiation, TME and adjuvant therapy, two main forms of radiation courses have been used based on their duration: long (LCRT) and short (SCRT) course radiotherapy [7]. LCRT delivers 45–56 Gy over a 5–6 week period with a concomitant sensitizing chemotherapeutic agent and a 6–12 week waiting period before TME, allowing for regression of the tumor. Several advantages have been associated with this strategy, including higher rates of colorectal anastomosis in low rectal tumors [8], reduction of local failures [8,9,10], and the possibility to identify good responders [11,12]. Conversely, during SCRT, a total of 25 Gy is administered in 5 fractions, followed by TME within 7 days. Similarly to LCRT, several phase III trials [13,14,15] have shown a significant reduction in local recurrence with SCRT.

Both neoadjuvant strategies have shown similar oncological results in terms of overall survival, local recurrence, and surgical complications [16]. However, due to the shorter time interval between RT and surgery, SCRT has been historically associated with lower rates of tumor response. Nonetheless, recent studies utilizing TNT with consolidation chemotherapy—thus allowing for longer time from SCRT to surgery—have shown good response rates [17,18]; however, direct, randomized data comparing the two RT regimens are currently not available [19].

Since up to 25% of LARC patients develop distant metastasis during follow up [20,21,22], the addition of systemic chemotherapy as a part of the NAT strategy has been proposed to decrease the risk of systemic failure, introducing the idea of TNT [23,24,25]. Additionally, several advantages have been associated with this strategy, including shorter ileostomy time and higher compliance to chemotherapy. More relevant to this review, TNT also enhances tumor response [24,26], particularly with a consolidation-based chemotherapy strategy as shown in the OPRA trial [27], providing more opportunities for organ preservation in selected patients.

## 3. Non-Operative Management and Patient Selection

Watch and wait (WW) is an organ preservation strategy for selected patients that experience a cCR, defined as the absence of detectable macroscopic tumor by clinical means after NAT, and is used interchangeably with NOM [2,28]. Despite the potential benefits of a WW strategy, many providers are reluctant to adopt it [29] because of the prior lack of standardization in response assessment criteria and the limitations of the published data prior to any integration of NOM into randomized trials. Moreover, there is an intermediate group of patients with near complete response (nCR) who demonstrate a significant tumor regression without achieving a true cCR [30]. Interestingly, up to 15% of patients with an nCR end up having a pCR [31,32] on pathologic examination. Therefore, a clear definition of cCR is paramount to increasing the adoption of WW, while maintaining oncologic safety; ideally, this would accurately select patients based on clinical assessment who would be found to have a pCR if they were to undergo surgical resection.

Initial evaluation should follow the standard rectal cancer work-up according to NCCN guidelines [1]. Endoscopic images of the tumor and rectal MRI at baseline represent key elements for subsequent assessment of treatment response [32]. After completion of NAT and thorough multidisciplinary discussion, patients with a cCR may enter a WW protocol with the understanding that this management does not represent a standard approach and that compliance with an intensive surveillance protocol is mandatory. Appropriate candidates for WW are often patients with mid-distal rectal adenocarcinomas, for whom alternatives are either an abdominoperineal resection or a low stapled/handsewn colorectal/coloanal anastomoses, which may negatively impact QoL due to permanent or temporary stoma and potential low anterior resection syndrome [33].

**Figure 1:**
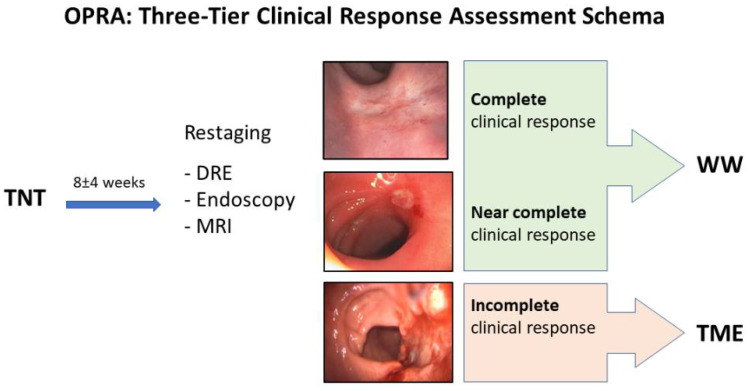
Memorial Sloan Kettering three-tiered response/regression schema.

In the OPRA trial, inclusion criteria were age older than 18 years, clinical stage II (T3-4, N0) or stage III (any T, N1-2) biopsy-proven rectal adenocarcinoma staged with MRI, a full colonoscopy, and computed tomography of the chest, abdomen, and pelvis [27]. Conversely, those with recurrent rectal cancer, evidence of distant metastasis at diagnosis, or history of pelvic irradiation were excluded. All patients with a complete or near-complete clinical response at re-staging (8 ± 4 weeks) were offered WW; patients with an incomplete response were recommended TME (Figure 1). OPRA is unique in that it prospectively applied the Memorial Sloan Kettering regression schema [2] to the patients, allowing response to inform the decision for WW or TME.

Patient selection based on pre-treatment characteristics is challenging, although some features, including <1 mm circumferential margin, extramural venous invasion, and extensive mesorectal/pelvic lymph nodes involved, are associated with lower cCR rates [34,35]. From a genetic profile perspective, mutations in *TP53* and *KRAS*, identified in ~70% and ~40% of rectal tumors [36,37], are associated with worse response to NAT, while mismatch repair deficiency tumors are overall associated with good response to NAT [38]. A relative contraindication is represented by ulcerated and circumferential tumors, which may be at risk of severe scarring and narrowing of the rectal lumen [39], preventing an appropriate endoscopic follow up.

While WW has been frequently adopted for older patients, it should also be discussed with younger patients as a viable treatment option. A recent report from the International Watch & Wait Database (IWWD) demonstrated no differences in disease-specific survival, local regrowth, and cumulative risk of distant metastasis between patients younger vs. older than 50 years of age [40].

Although not yet part of the NCCN guidelines, organ preservation remains a valid option in the context of optimal oncologic management of LARC, particularly when integrating patients’ inputs and desires. Gani et al. [41] reported that 83% of patients would opt for a WW strategy if they had a cCR in spite of higher rates of local regrowth, suggesting that they were willing to accept potentially worse oncological outcomes to avoid major surgical intervention. Additionally, in a survey of patients treated at a tertiary Canadian center, patients would accept a 20% absolute decrease in survival, while physicians would only accept a 5% absolute decrease in their patients’ overall survival [42].

## 4. Assessment of Response

The local assessment of tumor response after NAT is based on digital rectal exam (DRE), endoscopy, and magnetic resonance imaging (MRI). The combination of these three modalities predicts absence of tumor with a reported accuracy of 98% [32]. DRE should reveal a smooth, normal mucosa, although some minor irregularities or soft scar could be palpated. On endoscopy, cCR presents with a flat white scar, telangiectasia, and absence of both ulcer and nodularity [39]. Finally, MRI findings of cCR include a scar not thicker than the rectal wall, only dark T2 signal, and no visible lymph nodes or restricted diffusion.

As mentioned, one of the main challenges in the adoption of WW is the lack of uniform and reproducible criteria for tumor response, particularly for patients with nCR. In the attempt to standardize the definition of clinical response, the Memorial Sloan Kettering three-tiered response/regression schema has been developed and tested prospectively in the OPRA trial [27] (Figure 1). The three tiers are defined based on DRE, endoscopy, and MRI (T2W and DWI sequences) and classified as complete, near complete, and incomplete clinical response. The follow-up protocol included DRE, endoscopy, and CEA three times a year for the first 2 years, and twice a year for 3 additional years; rectal MRI was performed twice a year for 2 years and yearly thereafter; CT of the chest, abdomen, and pelvis was obtained annually.

Novel tools, including dynamic contrast-enhanced MRI, radiomics, molecular markers, and ctDNA have not been incorporated in current practice due to limited data, but provide provocative non-invasive endpoints that can be correlated with hard clinical endpoints of response for future investigation and validation [43,44,45,46].

## 5. Optimizing Tumor Response: The OPRA Trial

Improving rates of response to NAT is associated not only with better outcomes, but also with increased rates of organ preservation. Potential strategies include implementing a TNT strategy, increasing the interval period between NAT and surgery, and administering consolidation chemotherapy after RT rather than induction chemotherapy followed by RT.

The use of systemic chemotherapy to improve pCR to NAT has been initially proposed based on a single-institution phase II trial including 32 patients with LARC [47], which demonstrated the potential feasibility of selective elimination of preoperative LCRT. Subsequently, the CAO/ARO/AIO-04 German phase III randomized trial confirmed higher rates of pCR when oxaliplatin was added to fluorouracil-based NAT [48]. More recently, the PRODIGE 23 trial [49] randomized patients with LARC to either three months of neoadjuvant mFOLFIRINOX followed by LCRT followed by TME and three months of adjuvant chemotherapy or standard of care (LCRT + TME + six months of adjuvant FOLFOX). The experimental arm had significantly higher rates of pCR (28% vs. 12%, *p* < 0.001). Additionally, the RAPIDO trial [17] showed similar results in terms of response within a cohort of MRI-high risk LARC patients who were randomized to SCRT and CAPOX or FOLFOX followed by surgery vs. the standard arm (pCR = 28% vs. 14%, *p* < 0.001).

A longer interval from NAT to TME is associated with improved tumor response. The Timing of Rectal Cancer Response to Chemoradiation Consortium trial showed an increase in pCR rates from 18% to 25% when the average time from RT to surgery was increased from 6 to 11 weeks [4]. Additionally, an analysis of a National Cancer Database including LARC patients undergoing LCRT found that waiting >8 weeks between RT and surgery was associated with higher odds of pCR [50].

The consolidation chemotherapy based-TNT approach has emerged as the new paradigm to optimize tumor response and expand the opportunity for organ preservation. Initial reports of the phase II German trial CAO/ARO/AIO-12 comparing four cycles of FOLFOX before (induction) or after (consolidation) LCRT showed higher pCR rates in the consolidation arm (25% vs. 17%) [51]. Long term follow-up of this cohort showed consolidation chemotherapy to have similar disease-free survival, toxicity, QoL, or stool incontinence as induction therapy, and therefore recommended this sequence to be the preferred TNT algorithm if organ preservation is a priority [52]. Interestingly, using a similar consolidation approach but using SCRT versus LCRT followed by FOLFOX or CAPOX, the group at Washington University has shown a promising signal relative to organ preservation (TME-free survival at 2-years > 60%) [18]. Of note, they employed the same MSK regression schema for assessment of response and whether this will persist when integration into a randomized setting is unknown.

The OPRA trial [27] is a phase II multicenter randomized trial in which patients were assigned to either induction or consolidation TNT and then proceeded to WW or TME based on response (Figure 1). A total dose of 5000–5600 cGy was delivered to the primary tumor bed and regional pelvic nodes with concomitant capecitabine (825 mg/m^2^ twice a day orally) or continuous infusion fluorouracil (FU; 225 mg/m^2^/d), per the treating oncologist’s preference. Patients also received eight cycles of FOLFOX or five cycles of CAPEOX before (induction) or after (consolidation) CRT. Tumor restaging and surveillance was performed as per the protocol described in the previous paragraphs. Only those with an incomplete clinical response were recommended to undergo TME. Of the 324 eligible patients, 158 were randomized to the induction and 166 to the consolidation arm. The 3-year disease-free survival was identical between the two groups (76%) and was similar to the historical comparison (75%). The proportion of patients who preserved the rectum at 3 years in the intention-to-treat population was 53% for the consolidation and 41% for the induction group (*p* = 0.01); the proportion of patients who actually preserved the rectum was 60% and 47%, respectively (*p* = 0.02). Of note, almost 10% of patients undergoing TME had a pCR on final specimen examination. Lessons learnt:

- Patients with near-complete response at restaging can still be offered watch and wait with a close surveillance protocol;
- The rate of rectal cancer response to neoadjuvant therapy is much higher than previously thought and takes time to be achieved;
- Organ preservation is achievable in half of the patients with rectal cancer treated with TNT, particularly when consolidation chemotherapy is employed.

## 6. Oncologic Outcomes

One of the major uncertainties of a WW strategy is the long-term oncologic result [53]. Regrowth occurs in 25–30% of patients with a cCR [54], most of them during the first 2 years of follow up. Habr-Gama et al. have reported local regrowth rates ranging between 3% and 30% [31,55], with surgical salvage operations being feasible in over 90% of the cases [56,57]; the Memorial Sloan Kettering experience was similar, with >90% long-term pelvic control in our series of 113 patients with cCR managed by WW [58]. Among 880 patients who underwent WW after a cCR in the International Watch and Wait Database (IWWD) [54], the 2-year cumulative incidence of local regrowth was 25%; meanwhile, the 5-year overall survival and disease-specific survival were 85% and 94%, respectively.

A potential drawback of NOM may be a higher rate of distant metastasis after tumor regrowth. Data from a retrospective series of patients treated at Memorial Sloan Kettering Cancer Center suggest increased risk of distant metastases in patients with local regrowth as compared to those without local failure [58]. This pattern was also observed in data reported from the IWWD [54]; in patients with local regrowth, the incidence of distant metastasis was 18%, while in those without it was 5%. Jimenez-Rodriguez et al. also found a high rate of distant metastases (~50%) in the small number of patients (6%) treated with TNT by a single surgeon that actively performed WW [59] who experienced local regrowth.

In the OPRA study [27], the investigators identified a higher rate of tumor regrowth in the induction compared to the consolidation arm (40% vs. 27%), but this did not result in any detriment to survival as previously described. In fact, the overall rate of distant metastases was approximately 20%, without significant differences between patients who had surgery at re-staging vs. regrowth. Moreover, in the preliminary reports [60], the risk of tumor regrowths and distant failures were lower in patients with clinical complete response than in patients with near-complete response. Lessons learnt:

- Non-responders are at risk of both local and distant relapse, which may be higher than the average LARC patient, but is likely due to more aggressive biology;
- The more aggressive biology of non-responders should be taken into consideration when making surgical decisions;
- The grade of clinical response in patients offered selective WW has similar prognostic value as pathologic response in groups of all treated with TME;
- The grade of clinical response at restaging after TNT predicts both organ preservation and oncologic outcomes.

## 7. Functional Outcomes

The true rates of bowel, genitourinary, and sexual dysfunction after TNT and WW remain unknown. Although TME is associated with a significant impact on QoL, organ preservation after pelvic radiation may also affect patient-reported outcomes. In a retrospective review of the Memorial Sloan Kettering experience, 21 WW patients were matched 1:1 with 21 who underwent sphincter-preserving surgery [61]. Patients in the watch-and-wait arm reported better function on the overall scale and on all of the subscales. Similarly, a case-matched study comparing 47 WW patients with 41 patients who had NAT and TME showed that QoL was better in the WW cohort [62].

There is a need for prospective evaluation of patient-reported outcomes. The OPRA trial included as additional end points bowel, urinary, and sexual function and quality of life. The findings will soon be reported in a separate analysis.

## 8. Summary and Future Directions

The OPRA trial was the first prospective, randomized study to integrate WW into a TNT strategy aimed to increase tumor response rates. Although this trial demonstrated that organ preservation is achievable in half of rectal cancer patients treated with TNT, the real-world challenge remains to identify responders by clinical assessment. Building on the experiences of the TIMING [63], CAO/ARO/AIO-12 [51], RAPIDO [17], and OPRA [27] trials, Fokas et al. designed an ongoing randomized study investigating SCRT versus LCRT, each followed by consolidation chemotherapy and utilizing cCR and organ preservation rates as endpoints [64]. Moreover, the JANUS phase II rectal cancer study has recently received NCI approval and is awaiting final protocol approval before beginning enrollment (collaborative trial between The Alliance for Clinical Trials, NRG Oncology, and Southwest Oncology Cooperative groups—Smith JJ. Chair—personal communication). This is a randomized trial assigning LARC patients undergoing LCRT to consolidative mFOLFOX, CAPOX, or mFOLFIRINOX with cCR as the primary endpoint. Finally, in the subset of patients who are mismatch repair deficient or MSI-H, Cercek et al. [65] have an ongoing phase II study investigating the use of induction PD-1 blockade alone and its impact on cCR at 12 months for WW candidates. The preliminary results were recently published, with a 100% cCR in 12 patients followed for at least 6 months. Given these data, Ciombor et al. [66] have also proposed a trial for MMR-d patients using PD-1, CTLA-4 blockade, and SCRT to maximize response, and are integrating cCR as an outcome along with rates of pCR, disease-free survival, treatment-related toxicity, sphincter-preservation rates, and tumor regression grade.

## 9. Conclusions

In conclusion, to date, the optimal use of NOM for LARC lies in the context of a prospective trial with a strict protocol and objective assessment standards. NOM strategies should be part of a multidisciplinary discussion which balances physician concerns over disease recurrence and patient preference, potentially sacrificing some degree of survival in favor of organ preservation.
